# Durable Metallized Liquid Crystal Polymer Fibers Enable Flexible and Tough Electrical Heaters

**DOI:** 10.3390/polym17081087

**Published:** 2025-04-17

**Authors:** Yajie Zhang, Xinting Huang, Jiachi Zhou, Wenlin Liang, Xinxin Li, Chuang Zhu

**Affiliations:** Shanghai Frontiers Science Center of Advanced Textiles, College of Textiles, Donghua University, Shanghai 201620, China

**Keywords:** liquid crystal polymer fiber, fiber surface modification, electroless deposition, conductive fiber, fiber-shaped electrical heaters

## Abstract

Fiber-shaped electrical heaters with high flexibility and excellent adaptability make an ideal candidate for the application of wearable electronics but still suffer from low strength and poor durability. Herein, an all-in-one Joule-heating fiber capable of outstanding mechanical properties, good heating efficiency, and long-term stability is reported by using polymer-assisted metal deposition to firmly coat Cu nanoparticles on high-performance liquid crystal polymer (LCP) fibers. Taking advantage of LCP, the resultant fibers exhibit a satisfying temperature threshold (up to 200 °C) and immense strength (2.94 GPa). By virtue of dense and continuous Cu film, these fibers show low electrical resistance (5.51 Ω/cm) and an ultrafast response rate (12.6 °C·s^−1^) at low supplied voltages (0.5–3.5 V). Benefiting from the levodopa/polyethyleneimine interface design, such fibers maintain nearly constant resistance after repeatable bending, folding, and even washing (50 cycles). Based on the above-mentioned merits, a wearable patch with a Joule-heating function is knitted by using as-made fibers to offer therapeutic benefits for human body joints. This work demonstrates prospective potential for enriching the challenging applications of fiber-shaped electrical heating systems.

## 1. Introduction

Fiber-shaped electrical heaters offer significant advantages over photothermal heaters, particularly in terms of high-power output. Coupled with their inherently excellent flexibility and adaptability, these attributes make them highly desirable for the development of wearable thermotherapy devices [1,2,3,4,5,6]. To date, fiber-based electric heaters made from cotton [7,8,9], nylon [10], or polyester [11,12] can indeed deliver excellent electrothermal performance, but they still face certain limitations. For example, the strength of these fibers is poor because of their insufficient orientation and low crystallinity, limiting their applications in heavy-load conditions such as exercise and rehabilitation [13,14,15]. Alternatively, liquid crystal polymer (LCP) fibers present a research idea to satisfy mechanical demands, attributed to their high crystallinity and oriented molecular structure. To endow high-performance LCP fibers with electrothermal function, coating conductive nanoparticles on their surface represents a promising solution because conductive materials allow for the generation of Joule heat through external voltage as current passes through them, owing to the inelastic collisions between accelerated electrons and phonons [16,17,18]. Recently, graphene [9,19,20,21,22,23], carbon nanotubes [24,25,26,27,28], and MXene [29,30,31,32] have been widely investigated for depositing on fiber surfaces to fabricate fibrous electrical heaters. Nevertheless, it is difficult to achieve a uniform dispersion of these materials in organic solvents, which often form aggregated large particles, leading to inferior conductive coating quality.

Electroless deposition (ELD), an autocatalytic redox chemical reaction in which metal cations in a metal salt solution are reduced into metallic nanoparticles and coated on the catalytically active surface sites of the substrate at ambient environments, emerges as an ideal strategy to produce metallized fibers with well-established conductive coatings, attributed to advantages of low cost, power independence, and large-area production [33,34]. Despite this, metal films are easily delaminated from fibers because traditionally ELD catalysts are captured on the fiber surfaces via weak physisorption. Luckily, polymer-assisted ELD has been developed to add polymers as an adhesion layer to bridge the metal/fiber hierarchical structures [35,36]. Liu et al. [37] reported a versatile approach for preparing highly durable conductive metal layers via ELD onto cotton yarns modified with polyelectrolyte brushes, which not only play a role as an adhesive interfacial layer between metal nanoparticles and fibers via covalent bonds and other tethering forces but also enhance the uptake efficiency and selectivity of catalysts. Although the resultant conductive yarns provide robust electrical durability even after extensive cycles of stretching, bending, rubbing, and washing, the polymerization of these brushes entails an inert N_2_ protection and imposes complex steps. It is encouraging to note that Zhu et al. [38] utilized dopamine to self-polymerize at ambient environments via one potting to assist the ELD of nickel on fiber surfaces, and such metallized fibers could survive under harsh mechanical tests. However, the adhesion force of this method is poor as the uncontrolled spontaneous polymerization of dopamine generates large aggregates and forms a non-uniform polymer coating. And the stability of composite fibers in response to environmental changes cannot be ensured, as it is influenced by the quality of the coating and the interface adhesion. Indeed, establishing a highly adhesive polymer interface between metal films and fiber surfaces to initiate ELD in a low-cost and simple manner is still very challenging.

To address these challenges, we here propose a viable solution for fabricating high-performance conductive LCP fibers with durable electrothermal function and strong mechanical properties. The key enabling feature of this method is the introduction of a levodopa/polyethyleneimine (L-DOPA/PEI) copolymer as the designed interface via dip coating and subsequent catalyst-activated ELD, leading to a dramatic increase in the strong adhesion and high robustness of metallized LCP fibers. Moreover, the LCP-based conductive fiber could be heated by Joule heat while exhibiting superior heating performance with rapid response and a high temperature threshold. Particularly, by knitting as-made fibers into rib fabrics, the wearable parts could be used as therapeutic devices for the elbow, knee, and abdominal regions of human body. We believe this all-in-one design of fibrous electrical heaters enables the advancement of thermal management devices and heating systems in industry.

## 2. Materials and Methods

### 2.1. Chemicals and Materials

Levedopa (L-DOPA, 99%, CAS: 59-92-7, McLean, Shanghai, China), polyethyleneimine (PEI, 99%, CAS: 9002-98-6, McLean, Shanghai, China), and ammonium tetrachloropalladate ((NH_4_)_2_PdCl_4_, 36.5%, CAS: 13820-40-1, McLean, China) were employed. The above reagents do not need to be purified and can be used directly.

### 2.2. Polymer Interface Design

The pure LCP fibers were cleaned in acetone and DI for 30 min, respectively. The L-DOPA/PEI polymer bath was prepared by dissolving L-DOPA and PEI in a Tris-HCL buffer at a mass concentration of 1:1. Subsequently, the fiber was immersed in this solution for 6 h. Following this, each substrate was rinsed with deionized water and dried with a N_2_ gas stream.

### 2.3. Preparation of Cu@LCP Fibers by ELD

The polymer-modified fibers were immersed in a (NH_4_)_2_PdCl_4_ bath for 2 h, at a solution concentration of 0.005 Mm/mL. The catalyst, which physically adsorbed onto the fiber surface, was also washed with deionized water. The polymer-coated LCP fibers, exhibiting catalytic activity, were then passed through a freshly prepared ELD bath. The Copper plating solution consisted of 12 g/L NaOH, 13 g/L CuSO_4_·5H_2_O, 29 g/L NaKC_4_H_4_O_4_·4H_2_O, and 9.5 mL/L formaldehyde (CH_2_O).

### 2.4. Characterizations

The sample parameter sizes used for testing are shown in Table S1. Here, UV-vis spectroscopy (UV-3600I Plus, Shimadzu, Kyoto, Japan) was used to analyze the polymer solution, and FTIR tests (NEXUS-670, Thermo Nicolet, Waltham, MA, USA) proved that the polymer was successfully coated on the fiber surface. XRD measurements were made by the Escalab 250 Xi made of Brucker in Germany using a beam with a Cu Kα wavelength. Field emission SEM (S-4800, Hitachi, Tokyo, Japan) was used to observe fiber micromorphology during the copper deposition. The tensile properties were measured using a universal testing machine (ZQ990A, Zhiqu, Shenzhen, China) at a rate of 5 mm/min. The thermogravimetric analysis (TGA8000, Perkinelmer, Waltham, MA, USA) test was conducted using a thermogravimetric analyzer under a nitrogen atmosphere from room temperature to 800 °C, with a heating rate of 10 °C/min. The resistance signals of the fibers were collected by a universal electric meter (DMM6500, Keithley, Cleveland, OH, USA). For the Joule heating investigation, external voltages were applied to the electrical heaters by a power supply (2280S-32-6, Keithley, Cleveland, OH, USA) and the continuous temperature change signals were characterized by a thermocouple thermometer (TA612C, TASI, Suzhou, China). Finally, an infrared thermal imager (FLIR-TOOLS^+^, Flir Systems, Arlington, VA, USA) was used to present the pictures of the electrical heaters under different temperatures. The ambient temperature of the heater performance test was the indoor temperature during winter (11 °C).

### 2.5. Statistical Analysis

Statistical analysis was compiled on the means of the data obtained from at least three independent experiments using Origin2024b software. All values were expressed as the mean ± standard deviation (SD) of the individual sample. The sample size (n) numbers for each experiment are indicated in the figure legends.

## 3. Results and Discussion

### 3.1. Strategy for Fabricating Flexible Electrical Heaters

**Figure 1:**
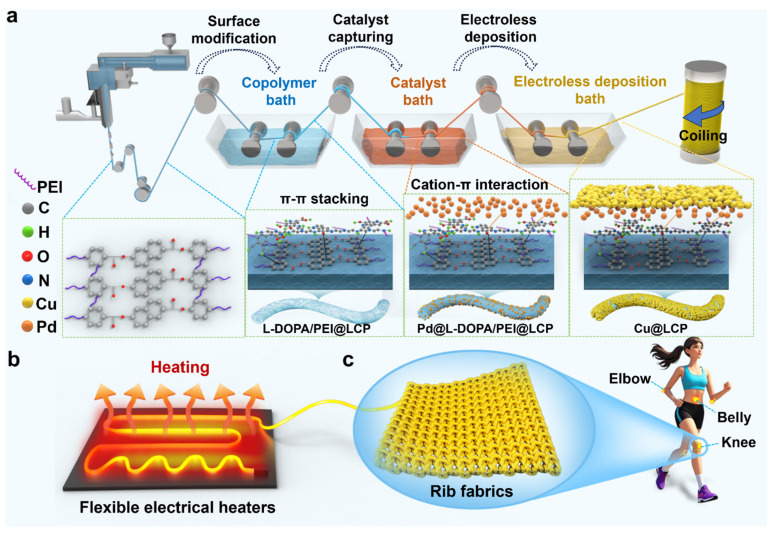
Strategy for fabricating flexible electrical heaters. (**a**) Schematic diagram of preparation process of the Cu@LCP fiber. (**b**) Schematic diagram of the fiber-based electrical heaters and their application. (**c**) Schematic diagram of fabrication as therapeutic heating devices in human bodies.

As presented in Figure 1a, the LCP fibers synthesized via melt spinning were subjected to a polymer-assisted ELD process, which involved three sequential steps to fabricate the Cu@LCP fiber composite. In the first step, a polymer interface was meticulously designed by incorporating a copolymerized L-DOPA/PEI system. This system effectively served as an adhesive, fostering strong interfacial adhesion between the copper (Cu) layer and the LCP fibers. The second step involved the introduction of a catalyst to enhance the catalytic activity of the ELD process, thereby facilitating the subsequent deposition of the copper nanolayer. In the final step, the Cu nanolayer was deposited onto the LCP fibers within the ELD bath at ambient temperature, completing the fabrication of the Cu@LCP composite. The resultant Cu@LCP fibers exhibited remarkable electrothermal performance, attributed to the Joule heating generated by the conductive copper sheath, which was efficiently transmitted to the LCP core. Figure 1b illustrates that individual Cu@LCP fibers could function as flexible electrical heaters. Furthermore, by knitting these versatile fibers into rib fabrics, wearable applications can be developed for use in various regions of the human body, including joints and the abdominal area (Figure 1c). These innovative textiles could serve as therapeutic devices, demonstrating excellent adaptability and comfort for users, thereby enhancing their potential in smart wearables and therapeutic applications.

### 3.2. Characterization of Interface Design and Surface Modification

**Figure 2:**
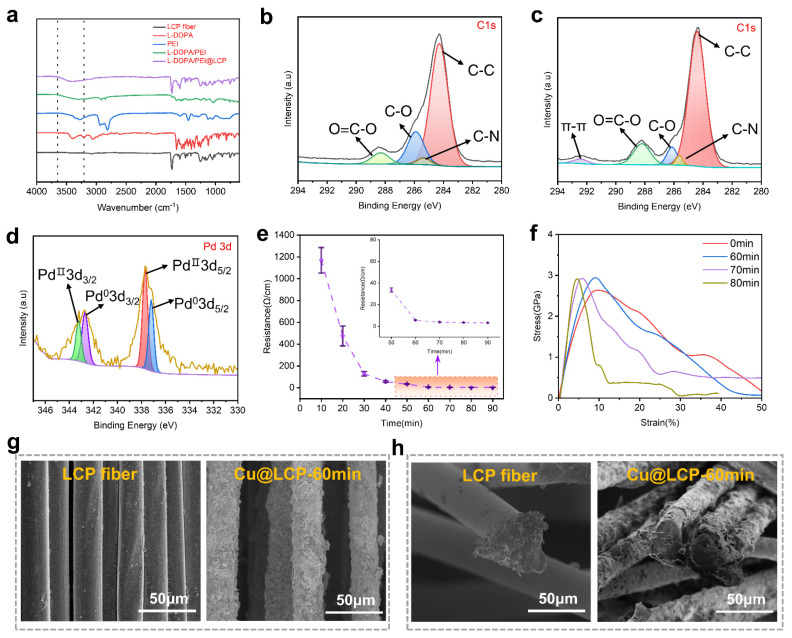
Characterization of interface design and surface modification. (**a**) FTIR spectra of LCP, L−DOPA, PEI, L−DOPA/PEI, L−DOPA/PEI@LCP. (**b**) XPS spectra of C1s of pure LCE fiber. (**c**) XPS spectra of C1s of L−DOPA/PEI−coated LCE fiber. (**d**) XPS spectra of Pd 3d of the Pd@L−DOPA/PEI@LCE fiber. (**e**) Surface resistance of Cu@LCE fibers along with time (*n* = 5). (**f**) Stress−strain curves of Cu@LCP fibers. (**g**) SEM images of the surface of pure LCP fiber and Cu@LCP fiber. (**h**) SEM images of the cross−section of pure LCE fiber and Cu@LCP fiber.

To establish a highly robust adhesion layer on the surface of the LCP fiber, a copolymer of L-DOPA and PEI was employed in the interface design. The copolymerization of L-DOPA and PEI at a 1:1 molar ratio resulted in a more homogeneous modification layer on the fiber surface, as demonstrated in previous studies [39]. As demonstrated in Figure S1, UV-visible spectroscopy confirmed the cross-linking interactions between L-DOPA and PEI. The analysis revealed a distinct absorption peak at 280 nm, corresponding to catechol groups, and an acromial peak at 300 nm, associated with dehydro-dopamine, present in both poly(L-DOPA) and the co-poly(L-DOPA/PEI) system. These findings indicate the occurrence of a Michael addition reaction of dopamine. Notably, the self-polymerization of poly(L-DOPA) exhibited a comparatively weak absorption peak at 400 nm, indicative of o-quinone formation. In contrast, the copolymerization of L-DOPA and PEI demonstrated a more intense absorption peak around 350 nm, attributed to the n-π* transition arising from the Michael addition mechanism. Figure 2a confirmed the successful grafting of the L-DOPA/PEI copolymer onto the surface of the LCP fibers, as evidenced by Fourier transform infrared spectroscopy (FTIR). The peak observed at 3500 cm^−1^ corresponds to the stretching vibration of hydroxyl groups. To further investigate the surface chemistry and interfacial bonding of the copolymer-grafted and catalyst-immobilized LCP fibers, X-ray photoelectron spectroscopy (XPS) was employed. In comparison to the C1s spectrum of unmodified LCP fibers (Figure 2b), the L-DOPA/PEI-coated LCP fibers exhibited the emergence of a new peak at 292.4 eV (Figure 2c), indicative of π-π stacking interactions between the L-DOPA/PEI copolymer and the LCP fiber substrate. The Pd 3d spectrum further elucidated the presence of reduced palladium (Pd⁰) at binding energies of 337.2 eV and 342.8 eV, along with chelated palladium (Pd^2+^) at 337.6 eV and 343.2 eV, which accumulated on the surface-modified LCP fibers subsequent to catalyst immobilization (Figure 2d). The presence of chelated Pb^2+^ ions is expected to enhance the catalytic activity of the electrochemical deposition process, while the reduced Pd atoms are anticipated to exhibit comparatively lower catalytic efficiency. Subsequent to the interface design and catalyst capture, a Cu nanolayer was deposited onto the LCP fibers within the ELD bath. This process caused the resistance to decrease and gradually stabilize with increasing ELD time. From Figure 2e, the surface resistance of Cu@LCP was 5.51 Ω·cm^−1^ after an ELD duration of 60 min. The tensile test results presented in Figure 2f indicate that the Cu@LCP fibers subjected to 60 min of ELD exhibited the highest tensile strength at 2.94 GPa and the greatest toughness at 65.53 MJ/m^3^. This enhancement in mechanical properties can be attributed to the formation of a relatively rigid coating layer due to the extended ELD duration, which consequently led to a reduction in tenacity and an increase in Young’s modulus, as further detailed in Figure S2. Figure 2g presents scanning electron microscopy (SEM) images illustrating that the pure LCP fibers exhibited a smooth surface, while the Cu@LCP fibers were characterized by a continuous and uniformly distributed Cu coating (Figure S3). The XPS spectra presented in Figure S4 further corroborated the successful deposition of a Cu layer onto the LCP fibers. The cross-sectional SEM analysis shown in Figure 2h further confirm the absence of significant voids between the Cu layer and the LCP fibers, thereby validating the superior interfacial adhesion achieved through the incorporation of the copolymer interface.

### 3.3. Characterization of Fiber Coating Quality

**Figure 3:**
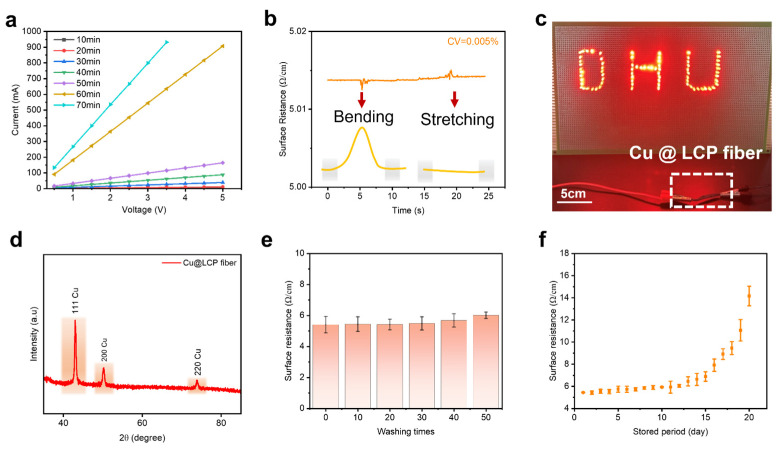
Characterization of electrical and stability properties of Cu@LCP fibers. (**a**) I−V curves of the conductive Cu@LCP fibers under ELD time of 10−70 min. (**b**) Real−time surface resistance upon repeated bending at an angle of 30° between the two ends of the fiber and stretching under the force of 10 N, 2 s. (**c**) Lighting of the “DHU” LED lamps via conductive Cu@LCP fiber. (**d**) XRD spectra of the conductive Cu@LCP fiber upon multiple voltage stimulus. (**e**) Surface resistance of Cu@LCP fibers along with washing times (*n* = 3). (**f**) Change in resistance of the Cu@LCP fiber with respect to the stored period at room temperature (*n* = 3).

To assess the electrical performance of the Cu@LCP fibers, external voltages were applied to the fibers subjected to varying durations of ELD. As the ELD time increased, the slope of the current–voltage (I-V) curve also increased, indicative of a reduction in resistance (Figure 3a). Notably, all I-V curves exhibited a characteristic linear relationship, which suggested excellent ohmic contact between the counter electrode and the conductive Cu nanolayer. Figure 3b demonstrated that the Cu@LCP fibers maintained a remarkably stable and consistent real-time resistance during both bending and stretching, with a coefficient of variation as low as 0.005%. Furthermore, the flexible conductive fibers successfully powered “DHU” LED lamps, maintaining steady brightness, as illustrated in Figure 3c. After multiple instances of sensing external voltage stimuli, the X-ray diffraction (XRD) spectrum of the Cu@LCP fibers (Figure 3d) revealed three distinct characteristic peaks at 2θ values of 43.00°, 50.22°, and 73.89°, corresponding to the Cu (111), Cu (200), and Cu (220) crystallographic planes, respectively. Importantly, no diffraction peaks associated with copper oxide were detected, indicating the exceptional stability of the Cu coating. Figure 3e showed that the surface resistance of the Cu@LCP fibers remained consistent after 50 cycles of washing with water followed by drying, demonstrating their durability under repeated mechanical and environmental stress. The stability of the metallic copper layer in Cu@LCP fibers was primarily attributed to the effective adhesion provided by the polymer interfacial bridging design between the fiber matrix and the copper layer. This design enhanced the fibers’ outstanding water resistance and antioxidative properties. Specifically, the antioxidant capability of L-DOPA within the copolymer layer neutralized free radicals presented in the water, while the hydrophilicity and adhesion strength of PEI further improved water resistance. Moreover, the copolymer layer created an effective barrier that isolated water molecules and oxygen from directly contacting the copper layer, thereby significantly reducing the likelihood of oxidation reactions. Furthermore, the sufficiently dense Cu layer effectively prevented self-oxidation. As a result, the performance of the Cu@LCP fibers remained stable during water wash cycling tests after being immersed in water for 0.5 h and subsequently dried in a vacuum oven. Additionally, as depicted in Figure 3f, the Cu@LCP fibers exhibited minimal changes in resistance over an extended storage period at a room temperature of 11 °C and humidity of 35%. To summarize, the remarkable electrical stability observed in the Cu@LCP fibers was believed to stem primarily from the innovative structural design implemented during the interface modification and coating processes. This design not only enhanced the interfacial adhesion but also contributed to the overall robustness and reliability of the conductive fibers in various applications.

### 3.4. Electrical Heating Performance of Cu@LCP Fibers

**Figure 4:**
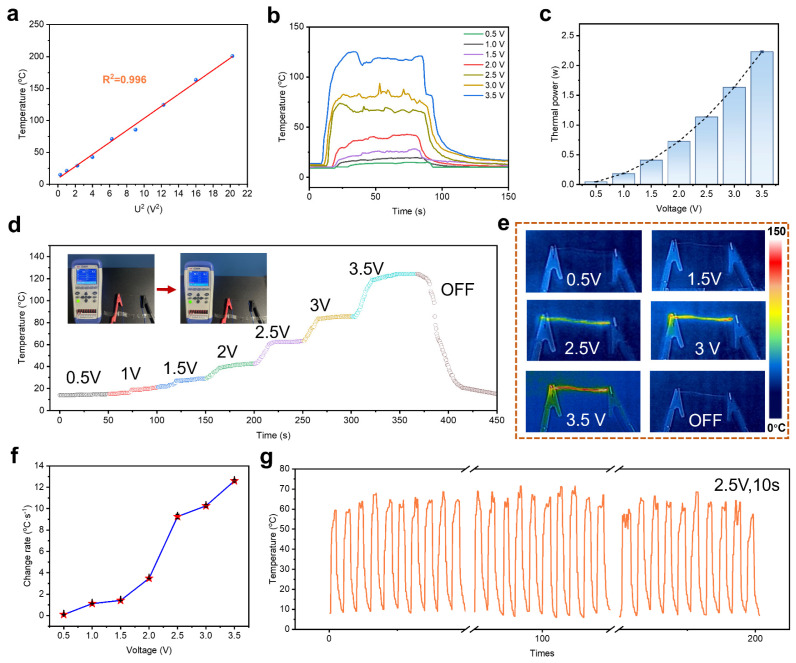
Characterization of electrical heating performance of Cu@LCP fibers. (**a**) Experimental data and linear fitting of saturation temperature versus U^2^. (**b**) The surface temperatures of the electrical heaters upon gradually changed voltages. (**c**) Thermal power of the electrical heaters upon gradually changed voltages (*n* = 3). (**d**) Time–surface temperatures curves of the electrical heaters with continuous different supplied voltages. (**e**) The infrared camera images of the Cu@LCP fibers upon different voltages of 0.5−3.5 V. (**f**) The heating rate of the Cu@LCP fibers under different voltages of 0.5−3.5 V (*n* = 3). (**g**) Long-term time-temperature curve at constant and repeated voltages of 2.5 V, 10 s.

For the Cu@LCP fiber, Joule heating generated by the Cu sheath could be effectively transferred to the LCP fiber substrate, thereby exhibiting the saturation temperature (Formulas (S1)–(S3)). During the Joule heating process, electrical current passing through a resistive material generates heat due to the resistance encountered by the electrons. The amount of heat produced is proportional to the square of the current and the resistance of the material, as described by Formula S4. The thermal resistance was critical for evaluating the electrical heating properties and practical applicability of the heaters. As illustrated in Figure S5, the Cu@LCP fiber exhibited high thermal degradation temperatures exceeding 500 °C, primarily due to the heat-resistant characteristics of the LCP substrate. This attribute was essential for accommodating the maximum operational heating temperatures of Cu@LCP fiber-based electrical heaters. Figure 4a presented a compelling linear relationship between the saturation temperature of the electrical heaters and the square of the supplied voltage, underscoring the reliability of the electrical heating performance. However, it is important to note that when the voltage exceeded 3.5 V, the rate of heat generation from the electric heater accelerated significantly, resulting in a substantial increase in energy output. This rapid escalation in temperature could lead to uncontrollable heating, posing a risk of fiber degradation. Consequently, this study emphasizes the steady-state temperatures achievable within a controlled voltage range of 0.5 to 3.5 V, as depicted in Figure 4b. The subtle fluctuations were attributed to thermal losses, the thermal inertia of the fiber matrix, and minor variations in resistance. As shown in Figure 4c, the steady-state saturation temperature (T_s_) increased with the increasing supplied voltage as more Joule heat power was generated from the electrical heaters (Figure S6). The experimental results in Figure 4d suggest that upon a gradual increase in supplied voltage, the flexible heater responded promptly with a continuous rise in temperature. Conversely, when the voltage was withdrawn, the temperature decreased back to room temperature, suggesting that these flexible electrical heaters possess significant potential for electronic applications with intelligently controlled heating (Figure S7). The illustration shows the experimental operation and the infrared camera images in Figure 4e, presenting the Cu@LCP fiber-based heaters with a uniform temperature distribution upon increased voltages under the above operation. More importantly, the results also indicate that the electrical heaters could reach their saturation temperature within a rapid timeframe of 3–30 s. For a quantitative assessment of the electrical heating capacity, Figure 4f revealed the rapid response of the Cu@LCP fiber-based heaters, with a speed of 12.6 °C·s^−1^. Furthermore, long-term evaluations of temperature stability under constant and repeated voltages are illustrated in Figure 4g and Figure S8, confirming the excellent reliability of the electrical heaters. This reliability is attributed to the innovative interface design and the dense copper nanolayer, which collectively enhance the performance and durability of the heating elements.

### 3.5. Applications of Cu@LCP Fiber-Based Heaters

**Figure 5:**
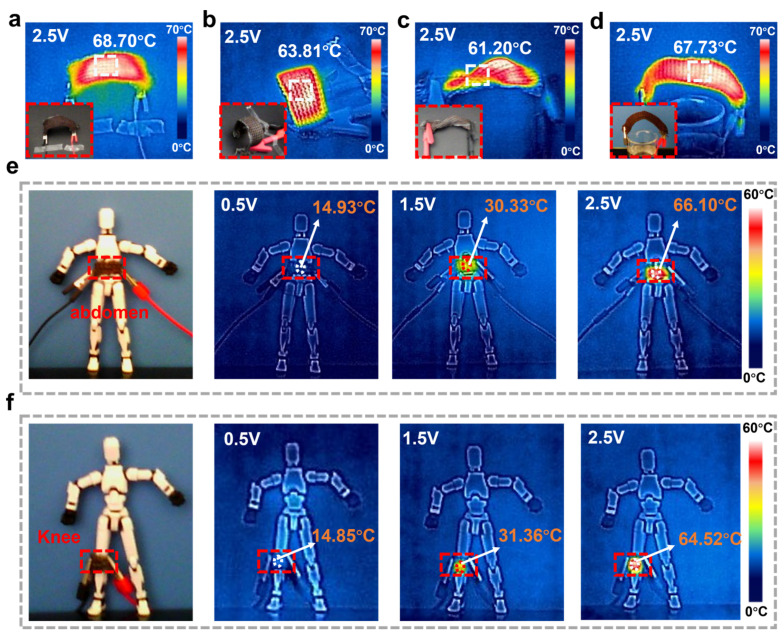
Applications of Cu@LCP fiber-based heaters. (**a**) Digital and infrared camera images of the electrical fabric heaters under small amplitude bending. (**b**) Digital and IR camera images of the electrical fabric heaters under bending of shape “C”. (**c**) Digital and IR camera images of the electrical fabric heaters under twisting. (**d**) Digital and IR camera images of the electrical fabric heaters after soaking in deionized water and drying. (**e**,**f**) Digital and infrared camera images of the electrical fabric heaters in wearable abdomen and knee thermotherapy with supplied voltages of 0.5, 1, and 1.5 V, respectively.

To further investigate the application capabilities, the Cu@LCP fibers were knitted into a rib fabric with a rib configuration of R = 2 (Figure S9). The transverse and longitudinal densities of the fabric were characterized as 18/5 cm and 13/5 cm, respectively, utilizing a manual flat knitting machine equipped with a seven-needle bed arrangement. Rib fabrics are renowned for their enhanced stretchability and elasticity compared to other knitted textiles, which makes them particularly suitable for applications requiring dynamic movements. Figure 5a–c illustrates digital and infrared imagery of the wearable rib fabric heater subjected to various bending and stretching maneuvers. These images revealed a consistent temperature distribution across the fabric, even under mechanical bending. Moreover, after immersion in deionized water followed by drying, the electrical fabric heater maintained a stable surface saturation temperature of approximately 67.7 °C (Figure 5d). Figure 5e,f provides further insights through digital and infrared images depicting the fabric heater in wearable thermotherapy, with applied voltages of 0.5 V, 1.5 V, and 2.5 V. Notably, thermal imaging demonstrated that at a low external voltage of 0.5 V, the electrical heaters affixed to the abdomen and knee exhibited temperature increases from an ambient temperature of approximately 9 °C to 14.93 °C and 14.85 °C, respectively. This change indicates a uniform temperature distribution along the heater’s surface, accentuating the precision of temperature control in the device. As the voltage was increased to 1.5 V and subsequently to 2.5 V, the temperatures of the fabric heaters rose to exceed 30 °C and 60 °C, respectively. These findings underscored the suitability of the Cu@LCP rib fabric heaters for integration into wearable devices designed to provide Joule-heated relief from stiffness and pain. In contrast to conventional thermal patches, the rib fabric exhibiting electrical heating capabilities offered superior adaptability to body contours and flexibility during movement. Furthermore, the coil sleeve’s uniform temperature distribution and its porous structure helped minimize heat buildup on the skin. As such, these knitted heaters represented a significant advancement in the development of therapeutic textiles, providing effective solutions for users requiring both comfort and performance in wearable heating applications.

## 4. Conclusions

In summary, the development of the all-in-one Joule-heating fiber presents a significant advancement in the field of wearable heating electronics. By integrating polymer-assisted metal deposition with high-performance liquid crystal polymer fibers, we have achieved a remarkable balance of mechanical strength, heating efficiency, and durability. The fibers exhibited impressive temperature thresholds and low electrical resistance, alongside a rapid response rate, making them highly suitable for various applications. Furthermore, the innovative levodopa/polyethyleneimine interface enhanced their resilience, ensuring stable performance under repeated mechanical stress and washing conditions. The successful demonstration of a wearable patch utilizing these fibers illustrated their potential for therapeutic applications, particularly in delivering localized heating benefits to human joints. This work opens new avenues for the deployment of fiber-shaped electrical heating systems in wearable technology, paving the way for further innovations in healthcare and beyond.

## Data Availability

The original contributions presented in this study are included in the article. Further inquiries can be directed to the corresponding authors.

## Supplementary Materials

The following supporting information can be downloaded at https://www.mdpi.com/article/10.3390/polym17081087/s1, Figure S1: UV diagram of poly(L−DOPA) and co−poly(L−DOPA/PEI) solution and at polymerization time of 6h, Figure S2: Characterization of mechanical properties of Cu@LCP fibers. toughness (a), Young’s modulus (b) of Cu@LCP fiber at different ELD time, Figure S3: SEM image and energy dispersive spectrometer (EDS) image of Cu@LCP fiber, Figure S4: XPS spectra of Cu 3d of Cu@LCP fiber, Figure S5: TGA curves of pure LCP fibers and Cu@LCP fibers, Figure S6: The temperature of the electrical heaters upon gradiently changed thermal power (*n* = 3), Figure S7: The temperature curves of Cu@LCP fiber−shaped heaters versus the time after removing the applied voltage, Figure S8: Changes of fiber surface temperature with heating times under different voltages for 10 s, Figure S9: The weaving diagram of the 1+1 rib structures; Formulas (S1)–(S4); Table S1: The sample size parameters used for the experimental tests.
