# Supplementary material for: Durable Metallized Liquid Crystal Polymer Fibers Enable Flexible and Tough Electrical Heaters
Source: Polymers (Basel). 2025 Apr 17;17(8):1087. doi: 10.3390/polym17081087 (PMC12030729; doi:10.3390/polym17081087)
Supplement: Supplementary file 1 [file polymers-17-01087-s001.zip › polymers-3592038-supplementary.pdf]

Supplementary Information

Durable metallized liquid crystal polymer fibers enable flexible and tough electrical heaters

Yajie Zhang[1], Xinting Huang[1], Jiachi Zhou[1], Wenlin Liang[1], Xinxin Li[1], Chuang Zhu[1,]*

[1] Shanghai Frontiers Science Center of Advanced Textiles, College of Textiles, Donghua University, Shanghai 201620, China

*Corresponding Author: Chuang Zhu (chuang.zhu@dhu.edu.cn)

Supplementary Figures

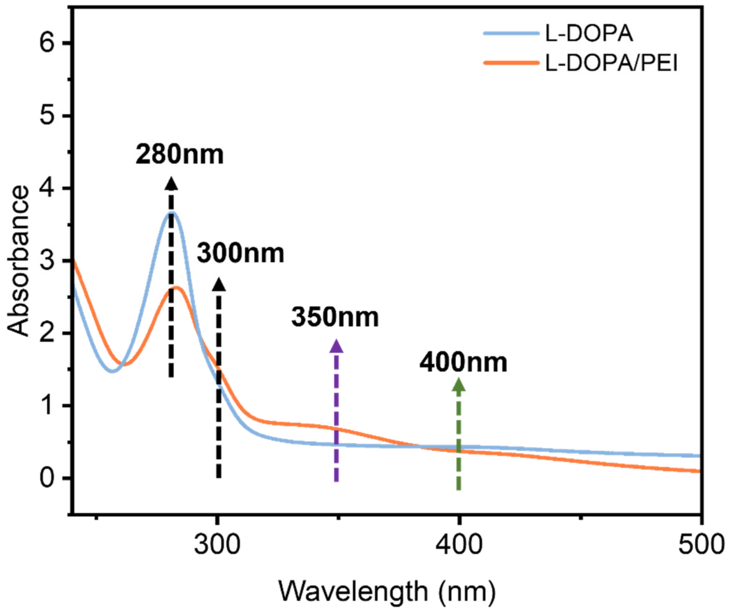

Figure S1. UV diagram of poly(L-DOPA) and co-poly(L-DOPA/PEI) solution and at polymerization time of 6h. The figure shows the absorption peaks of poly(L-DOPA) and co-poly(L-DOPA/PEI) solutions at different intensities around 280nm (catochol groups), 300nm (dehydrodopamine) respectively. From the picture, a stronger absorption peak around 350 nm of co-poly(L-DOPA/PEI) was shown, in contrast to the weak absorption peak at 400 nm (o-quinone) of poly(L-DOPA).

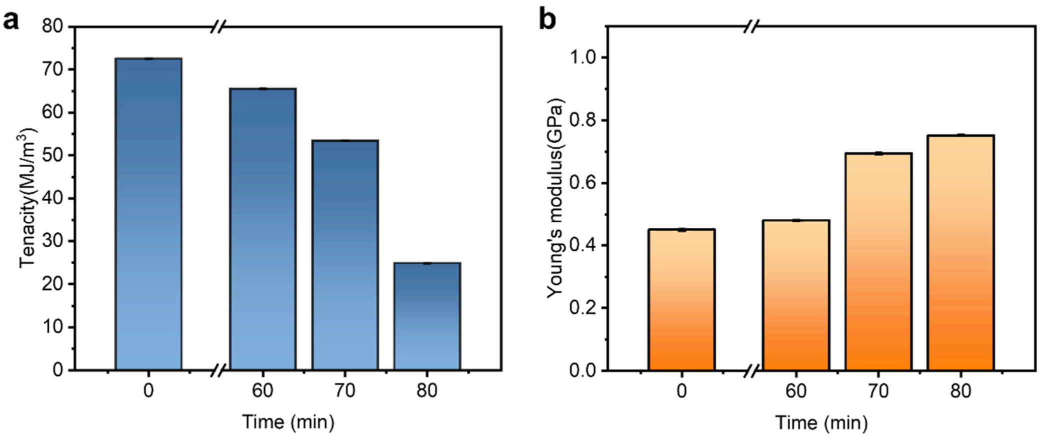

Figure S2. Characterization of mechanical properties of Cu@LCP fibers. toughness (a), Young's modulus (b) of Cu@LCP fiber at different ELD time. With the increased ELD time from 60 to 80 min, the tenacity decreased from 65.53 to 24.88 MJ/m^3 corresponding to the Young's modulus increasing from 0.48 to 0.75 MPa. By analyzing the conjunctive results, 60 min was chosen as the optimal ELD time.

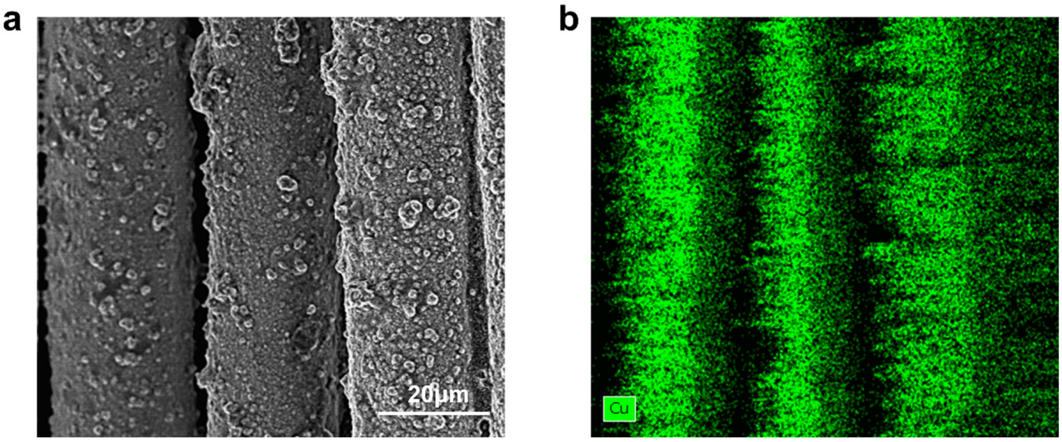

Figure S3. SEM image and energy dispersive spectrometer (EDS) image of Cu@LCP fiber. The image demonstrated that the coating layer on LCE fiber was Cu.

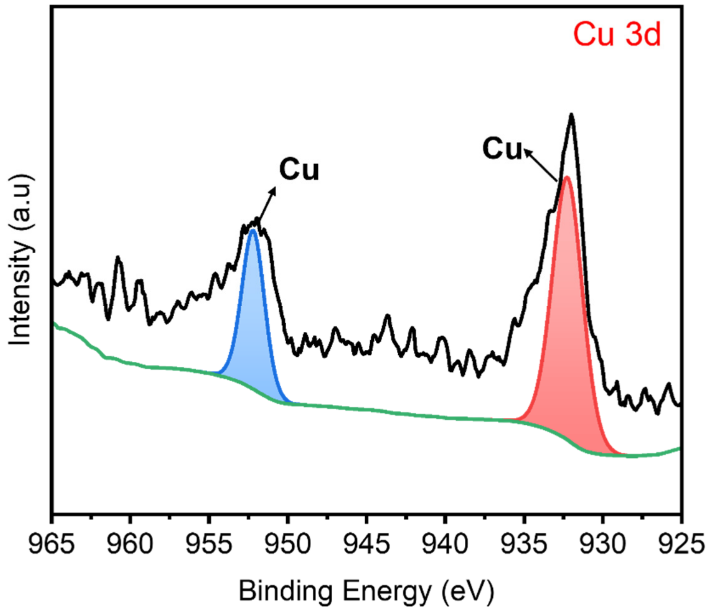

Figure S4. XPS spectra of Cu 3d of Cu@LCP fiber. The image confirmed the successful coating of the Cu layer on the LCP fiber.

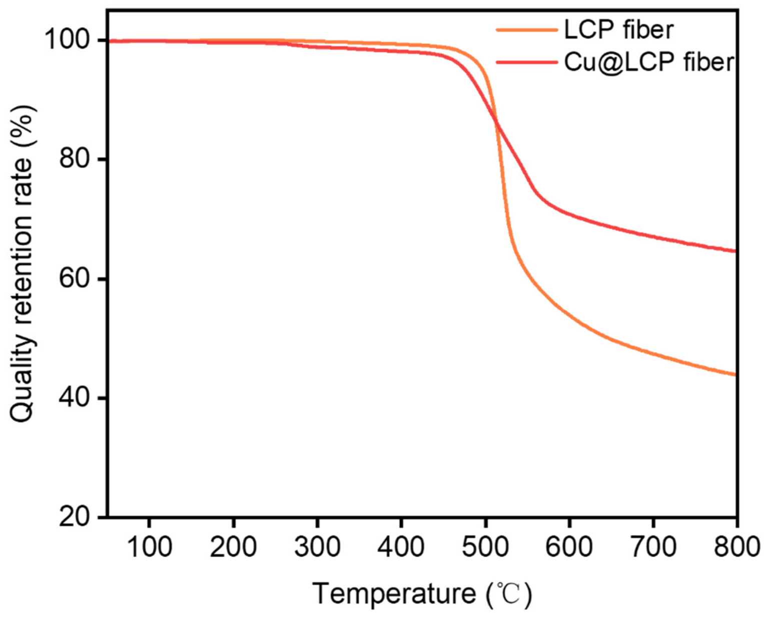

Figure S5. TGA curves of pure LCP fibers and Cu@LCP fibers. The fibers were treated under N2 from room temperature to 800°C and the TGA image clarified the thermal stability of pure LCP fibers and Cu@LCP fibers.

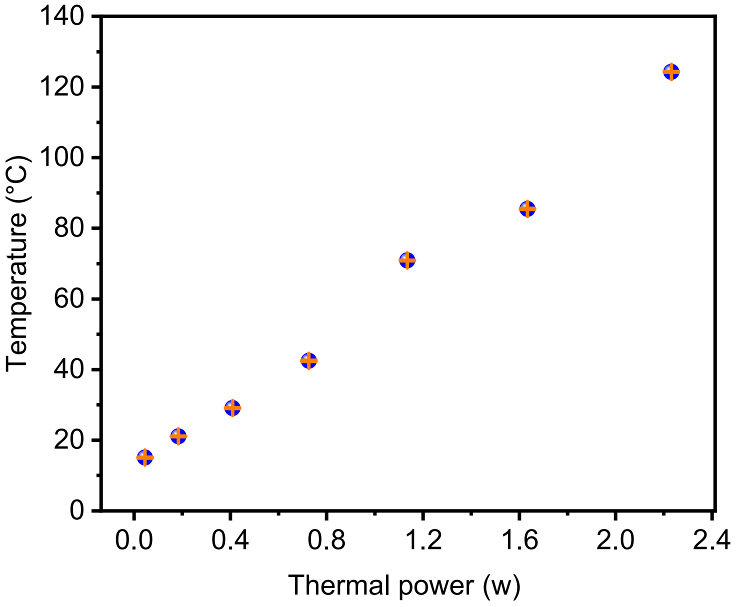

Figure S6. The temperature of the electrical heaters upon gradiently changed thermal power (n=3). As the thermal power generated by Joule heating increased, the surface temperature of the fibers gradually rised. The thermal power was associated with both the applied voltage and the surface resistance of the fibers, which was consistent with the conclusions derived from the theoretical formulation.

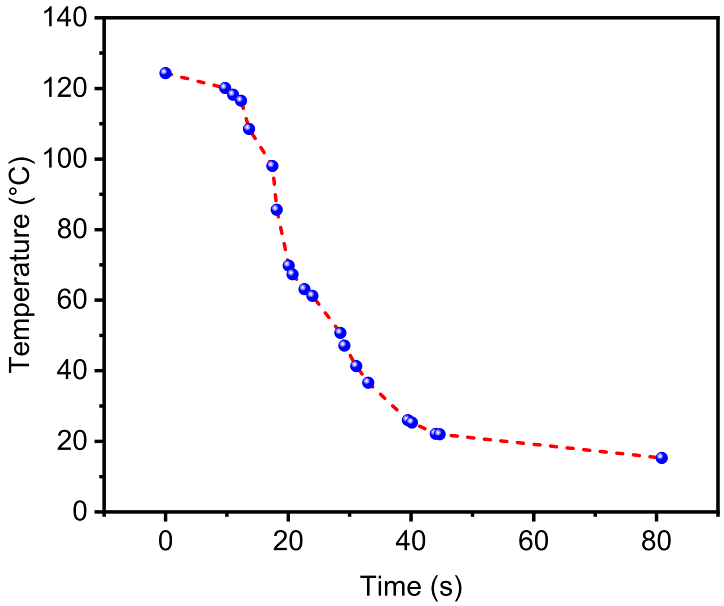

Figure S7. The temperature curves of Cu@LCP fiber-shaped heaters versus the time after removing the applied voltage. Upon the removal of the applied voltage, the temperature variation curve of the Cu@LCP fiber heater illustrated distinct cooling characteristics. Specifically, after the voltage was withdrawn, the surface temperature of the fiber gradually decreased, with the initial rate of temperature declined notably exceeding that observed in the later stages. Approximately 80 s later, the fiber's temperature approached its initial state. This phenomenon revealed insights into the electrical heating and subsequent thermal dissipation behavior of the material.

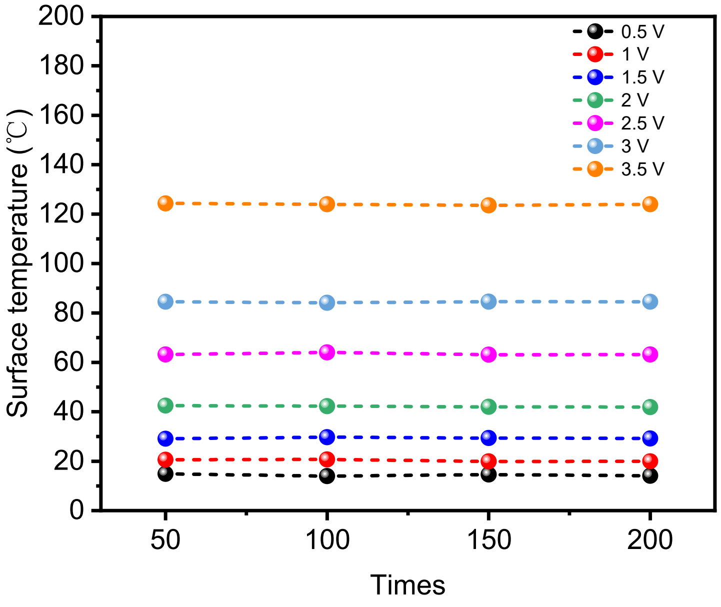

Figure S8. Changes of fiber surface temperature with heating times under different voltages for 10 s. When repeated heating 200 times, the fiber electric heater could still reach a stable surface saturation temperature under voltage stimulation, which proved the durability and repeatability of the fiber electric heater.

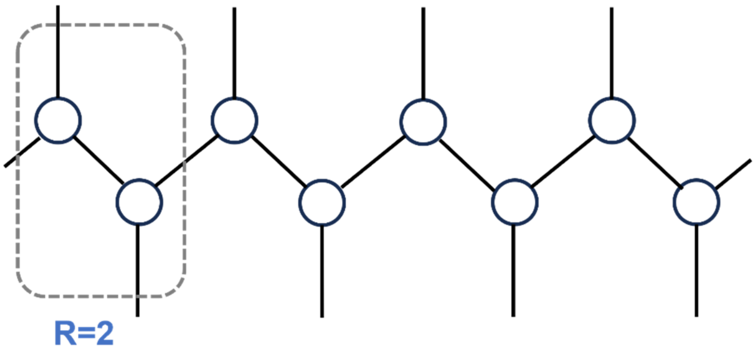

Figure S9. The knitting diagram of the 1+1 rib structures. The 1+1 rib knitting structure was formed by alternating a column of front loops and a column of back loops. A complete organization was defined as the minimum repeating unit, which consists of one front loop and one back loop.

Supplementary Formula

To comprehend the heating behavior mechanisms, we performed a thermodynamic analysis of the heating performance of Cu@LCP fiber-based electrical heaters. By applying the energy balance principle, we established that the surface temperature of the heaters reaches equilibrium when the power generated by Joule heating equals the losses from conduction, convection, and radiation. Thus, the surface temperature can be calculated using the following formula:

$$\frac{U^2}{R} = mc\frac{dT}{dt} + hS\Delta T \quad \text{(S1)}$$

Where U is the supplied voltage, R is the surface resistance and m is the mass of the electrical heaters, c is the specific heat capacity, h is the convective heat-transfer coefficient, S is the area of the electrical heater, and ΔT is the difference between the surface temperature and the initial ambient temperature. The following formula is obtained through the integration and deformation of the above integral equation:

$$\Delta T = \frac{U^2}{RhS}(1 - e^{-\left(\frac{hs}{mc}\right)t}) \quad \text{(S2)}$$

Considering that t =0, the saturation temperature (T_s) is equal to the initial ambient, which could be acquired as following:

$$T_s - T_0 = \frac{U^2}{RhS} \quad \text{(S3)}$$

As we can see, T_s of the electrical heaters is only influenced by the applied voltage, surface resistance and convective heat-transfer coefficient.

During Joule heating process, electrical current passing through a resistive material generates heat due to the resistance encountered by the electrons. The amount of heat produced is proportional to the square of the current and the resistance of the fiber-based electrical heaters, described by the formula:

$$Q = I^2 R \Delta t \tag{S4}$$

Where Q is the amount of heat produced, I is the current passing through the fiber, R is the resistance and Δt is the interval between the applied voltage and the withdrawn voltage.

Supplementary Table

Table S1: The sample size parameters used for the experimental tests.

Parameters	Description	Value (Unit)
L_{LCP}	Length of the LCP fiber	10 cm
$R_{Cu@LCP}$	Surface resistance of the LCP fiber	5.51 Ω
R	Fabric configuration of the Cu@LCP rib	2
P_A	Transverse density of the Cu@LCP rib	18/5cm
P_B	Longitudinal density of the Cu@LCP rib	13/5cm
S_1	Fabric area for bending test	10*4cm
S_2	Fabric area for bending test	3*3cm
